# Dynamic Stabilization for Challenging Lumbar Degenerative Diseases of the Spine: A Review of the Literature

**DOI:** 10.1155/2013/753470

**Published:** 2013-04-15

**Authors:** Tuncay Kaner, Ali Fahir Ozer

**Affiliations:** ^1^Department of Neurosurgery, School of Medicine, Istanbul Medeniyet University, 34730 Istanbul, Turkey; ^2^Department of Neurosurgery, School of Medicine, Koc University, 34365 Istanbul, Turkey

## Abstract

Fusion and rigid instrumentation have been currently the mainstay for the surgical treatment of degenerative diseases of the spine over the last 4 decades. In all over the world the common experience was formed about fusion surgery. Satisfactory results of lumbar spinal fusion appeared completely incompatible and unfavorable within years. Rigid spinal implants along with fusion cause increased stresses of the adjacent segments and have some important disadvantages such as donor site morbidity including pain, wound problems, infections because of longer operating time, pseudarthrosis, and fatigue failure of implants. Alternative spinal implants were developed with time on unsatisfactory outcomes of rigid internal fixation along with fusion. Motion preservation devices which include both anterior and posterior dynamic stabilization are designed and used especially in the last two decades. This paper evaluates the dynamic stabilization of the lumbar spine and talks about chronologically some novel dynamic stabilization devices and thier efficacies.

## 1. Introduction

Today, low back pain is one of the most important problem in decreasing the quality of life as a result of lumbar disc degeneration [1–4]. It is thought that the origin of low back pain results from degenerative intervertebral disc and facet joints. Segmental instability significantly contributed to lower back pain. Instability associated with intervertebral disc degeneration is represented first by Knutsson in 1944 [5]. Knutsson also described the abnormal flexion-extention slipping in X-ray along with disc degeneration and told that segmental instability should be if sagittal slipping is greater than 3 mm in dynamic X-ray. Degeneration process of the lumbar spine and pathology of discogenic pain were described by Kirkaldy-Willis and Farfan in 1982 [2]. They explained that degenerative instability of the spine began primarily with disc degeneration which contains dehydration of intervertebral disc along with decrease in tension of the annulus fibrosis. It is followed by decrease of disc height, and then this process continues with hypertrophy of the facet joint and ligamentum flavum. At the end spinal stenosis and degenerative spondylolisthesis, which have caused low back pain, occur. Besides, Frymoyer and Selby revealed the concept of primary and secondary instabilities and put the degenerative disc disease, degenerative spondylolisthesis, and degenerative scoliotic deformities into the group of primary instability [6, 7]. Panjabi also well defined the term instability that leads to a pain, pathological movement, deformities, and neurological inability [8]. Afterwards, Benzel splitted the chronic instability to two groups which were glacial instability and dysfunctional segmental motion [9]. According to Benzel the commonest sample for glacial instability is spondylolisthesis which has been seen as degenerative, isthmic, and iatrogenic and for dysfunctional segmental motion is degenerative disc disease.

Within last century the surgical treatment of disc-related pain began with discectomies and decompressions. First lumbar discectomy surgery has been performed by Mixter and Barr in 1934 [10]. However, they could not obtain the relief of chronic low back pain after their disc excision operation. Afterwards, radical discectomies and subtotal discectomies have been performed commonly, but good clinical results have not been obtained, and lower back pain and continuous sciatica as high as 40% insisted [11–14]. Insisting low back pain and sciatica after discectomy procedures have been engaged to segmental instability, and the concept of chronic and degenerative instability has been suggested and then developed within years [2, 5–9]. Some studies showed that decompression with fusion (posterolateral or interbody) meaningfully improved patient outcome compared to decompression alone [15–18]. Fusion was carried out to cease the motion for stopping the pain in degenerative disorders of the lumbar spine [19], but every time the achievement could not been arrived because to fuse the moving spine was hard [20, 21]. Later, internal fixation systems have been discovered by pioneers like Harrington, Dick, Magerl, and Roy Camille and commonly used with fusion [22–24]. Rigid pedicle screw fixation of spine improves the ratio of successful fusions according to some biomechanical studies [25].

Rigid internal fixation and fusion have been currently the mainstay for surgical treatment of degenerative diseases of the spine over the last 4 decades. In all over the world the common experience was formed about fusion surgery. Although successful radiological results up to 100% associated with fusion reported, this results were not compatible with successful clinical outcome regarding pain alleviation [15, 26, 27]. Satisfactory results of lumbar spinal fusion appear completely incompatible and ranged from 16% to 95% with an average of 70% according to a meta-analysis study evaluated systematically [28]. Rigid spinal implants along with fusion also cause increased stresses of the adjacent segments, and adjacent segment degeneration, which is well known, is formed [29–34]. In addition fusion surgery has some important disadvantages such as donor site morbidity including pain, wound problems, infections because of longer operating time, pseudarthrosis, and fatigue failure of implants [35–38].

**Figure 1:**
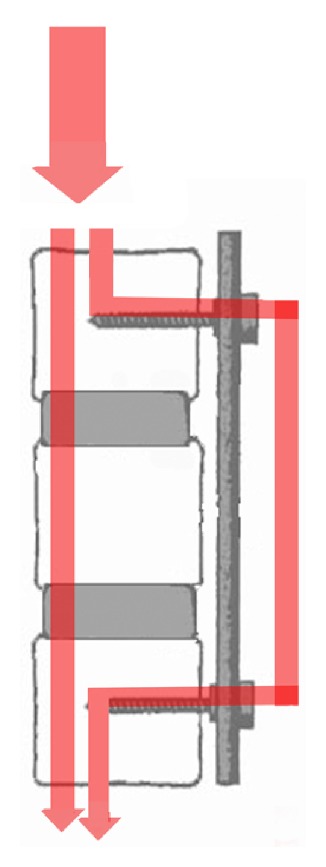
Posterior dynamic stabilization provides more physiological load transmission between anterior and posterior components of the spine.

The search for alternative spinal implants was supported with time on unsatisfactory outcomes of rigid internal fixation along with fusion. The main aim was to avoid the opposed effects of rigid implants on the stabilized and adjacent segments, to prevent the implant failure and to provide reduced-stress shielding, and finally to develop a system that permits increased load sharing and controlled motion without cutting off the stability [39]. İntervertebral disc actually has a isotropic architecture like a fluid-filled ball, but it changes as intervertebral disc is degenerated. Isotropic properties and load transmission of the intervertebral disc alter depending on disc degeneration [40, 41]. The “stone-in-the-shoe” phenomenon explains the postural pain pattern in patients suffering from lumbar disc degeneration because pattern of loading is related to pain generation in the degenerated spine which alters one patient to another [41, 42]. Dynamic stabilization intends to eliminate the pain by delivering the weight with more physiological load transmission between anterior and posterior components of the spine while attempting to maintain the motion and to control abnormal movement in the spinal segment [42, 43] (Figure 1). It is supposed that soft or semirigid stabilization systems restore normal functions of the spine unit and protect the adjacent segments [43, 44]. Dynamic stabilization of spine has been classified by several authors [4, 45, 46]. Today in market different both anterior and posterior dynamic stabilization devices of spine are found. Various biomechanical and clinical studies were done about dynamic stabilization systems of spine. Recently finite element studies have begun more and more on these systems. Some clinical studies concerning dynamic screws and dynamic rods have been revealed [37, 44, 47–49]. Recently it is thought how we can do the dynamic stabilization systems which is close to more physiological pattern.

## 2. Indications of Dynamic Stabilization of Spine

**Figure 2:**
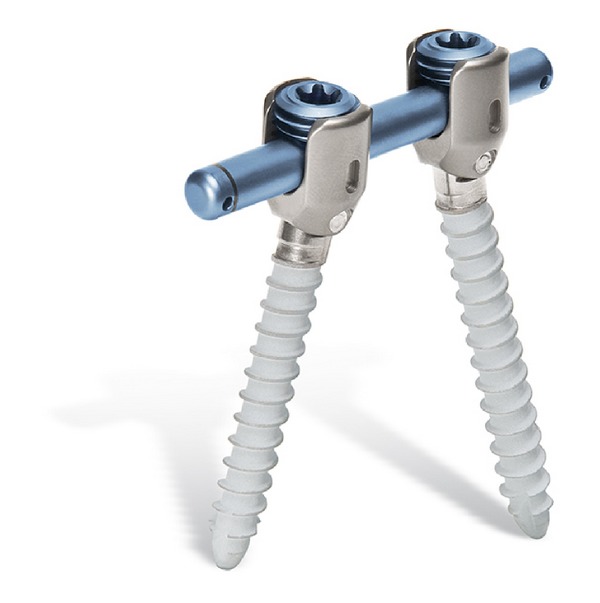
Cosmic posterior dynamic system.

Strempel et al. revealed the indications for dynamic stabilization with cosmic (semirigid posterior dynamic system including dynamic screw and rigid rod) [50] (Figure 2). These are symptomatic lumbar stenosis, chronically recurrent lumbago in the case of discogenic pain and facet syndrome, recurrent disc herniation, in combination with a spondylodesis, and extention of an existing spondylodesis in the case of a painful adjacent level degeneration. Strempel suggested that cosmic posterior dynamic stabilization should only be used for a maximum of three segments.

Khoueir et al. revealed the indications for posterior dynamic stabilization in 2007 [45]. The indications included controlled motion in the iatrogenically destabilized spine, increased anterior load sharing to augment interbody fusion, protection and restoration of degenerated facet joints and intervertebral discs, in combination with anterior motion preservation for 360 circumferential motion segment reconstruction, adaptation of stabilization techniques to the aging spine, and the prevention of fusion-related sequelae.

Kaner et al. described a new classification system about dynamic stabilization of spine [4]. They reported the indications related to posterior dynamic stabilization systems. They included degenerative spinal instability (disc degeneration, facet degeneration, and degenerative spondylolisthesis), iatrogenic instability following discectomy/decompressive laminectomy, increased anterior load shared to augment interbody fusion, stabilization of a painful adjacent segment degeneration, adjacent to fusion, complement TDR to achieve anterior disc replacement, and second recurrent of a disc herniation. Lomber disc herniations which were graded III and IV are based on Carrage Classification system [51]. The reherniation rate is quite high in these groups (%27) and if surgery is supported with a dynamic system, reherniation rate significantly decreases [51, 52]. They also reported the indications related to interspinous distraction devices which are central spinal canal stenosis with neurogenic claudication, foraminal stenosis with radicular symptoms, and facet joint disease, in one- or two-level stenosis in patients over 50 years. As third the indications related to anterior disc prosthesis are patients between 18–60 ages (optimally below age 50 years), single level or two levels, pain due to symptomatic degenerative disc disease, absence of facet joint degeneration changes, existence of intervertebral disc height of at least 4 mm, nonradicular leg pain or back pain, postlaminectomy syndrome, and patient with positive discogram [4].

## 3. Anterior Dynamic Stabilization

### 3.1. Anterior Disc Prosthesis

Lumbar degenerative disc disease is the commonest indication of lumbar disc prosthesis. Patients selected for anterior disc prosthesis should be proven to have discogenic pain or segmental instability, or both, without advanced degenerative arthritis. However, spinal pathology should be demonstrated by routine radiological evaluations including upright X-rays, magnetic resonance imaging (MRI), and provocative discography. A lumbar disc prosthesis may be used to restore discogenic instability and improve the discogenic pain after discectomy procedure or at places of previous discectomy.

**Figure 3:**
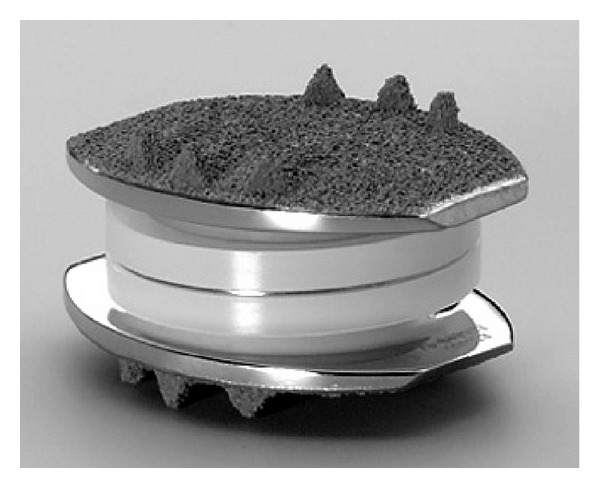
Charite III artificial lumbar disc prosthesis.

**Figure 4:**
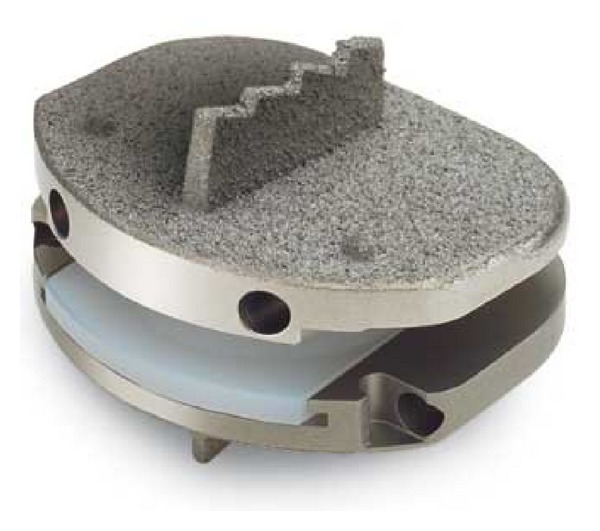
ProDisc artificial lumbar disc prosthesis.

The history of arthroplasty of spine begins with Paul Harmon, from 1959 to 1961. He used vitallium balls through an anterior approach to stabilize vertebral segments to assist fusion and realized that some of them could be work well as stand-alone stabilizers (the first disc arthroplasty) [53]. Second specialist who is Ulf Fernstrom, from 1962 to 1972, used the steel ball arthroplasty of the spine via posterior lumbar approach [53]. The first published article about disc arthroplasty belongs to Fernström in 1966 [54]. Later Reitz and Joubert (1964) [55] and Mc Kenzie (1971) [56] used steel ball arthroplasties. These specialists imagined and tried earlier disc arthroplasty procedures and motion preservation surgery. In this surgeries ball has an extremely low surface contact area on initial implantation which may lead to subsidence. Pain, disc height loss, loss of motion, and many times fusion were seen in many cases depending on subsidence. The usage of Fernstrom has been left afterwards. The first modern prosthesis, CHARITE, was designed and first implanted by Büttner-Janz et al. in 1984 [57]. First Charite disc prosthesis had a polymer core which is either floating, unconstrained, and between two concave end plates. Within years Charite III (DePuy Spine) was accepted in its final form and certified by the FDA in October of 2004 [58] (Figure 3). Today it is widely used clinically. The semiconstrained disc prosthesis design, ProDisc (Synthes), was implanted first by Rousseau et al. in 1990 [59] (Figure 4). CHARITE and ProDisc artificial disc prostheses have the architecture of hard plates/hard core designs. There are several artificial disc prosthesis having different design. These are hard plates/soft core, screw-In dowel, spring and piston, and complex mechanical/vertebral body replacement [60]. Lumbar disc prosthesis is recommended, L4-L5 and L5-S1 disc levels, but especially L4-L5 disc level is ideal for maintaining the motion. This approach is more logical with regard to the philosophy of motion preservation. Satisfactory outcomes are seen after anterior disc prosthesis if it covers surgical indications. Some studies also revealed good clinical results [61, 62].

### 3.2. Nucleus Replacement

**Figure 5:**
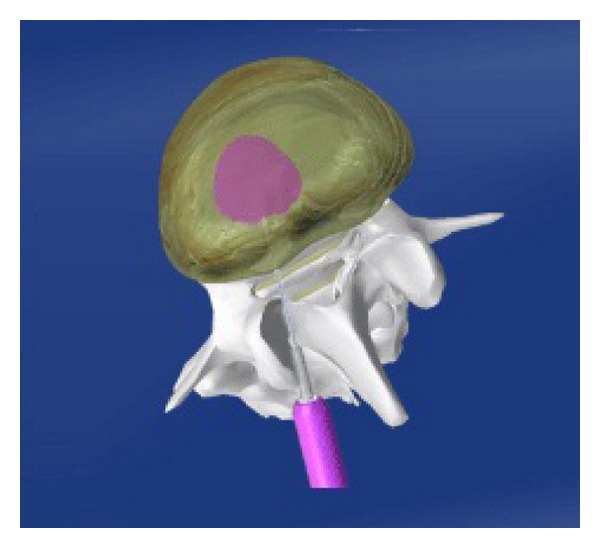
Nucleus replacement, NuCore and BioDisc.

**Figure 6:**
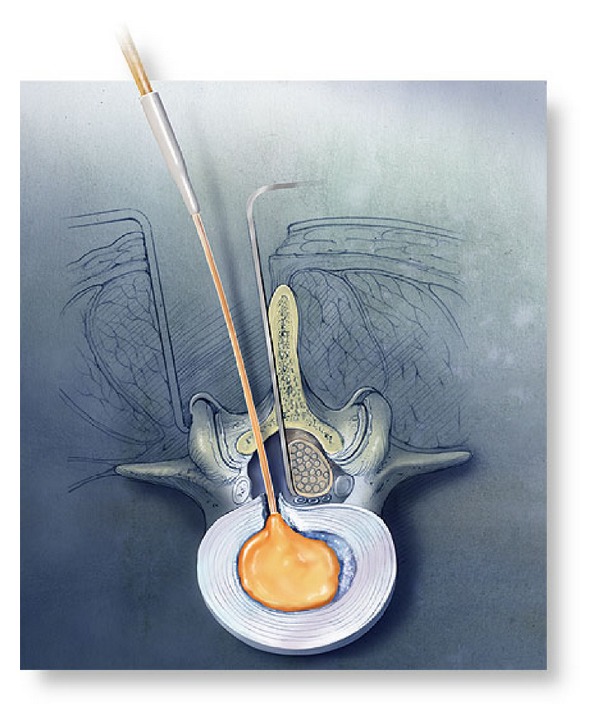
Nucleus replacement, NuCore and BioDisc.

**Figure 7:**
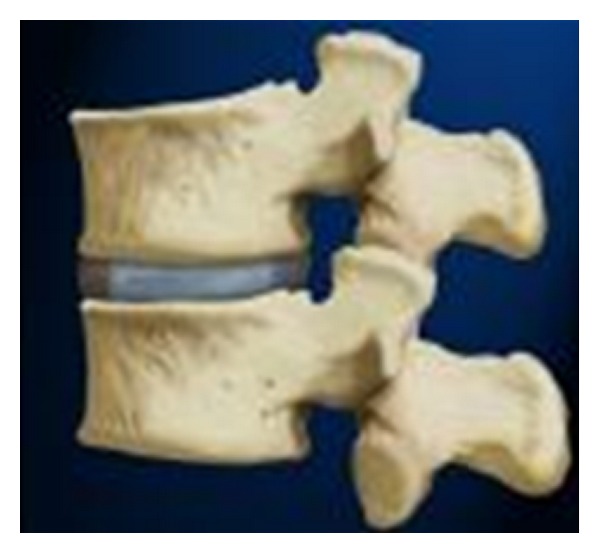
Nucleus replacement, DASCOR.

**Figure 8:**
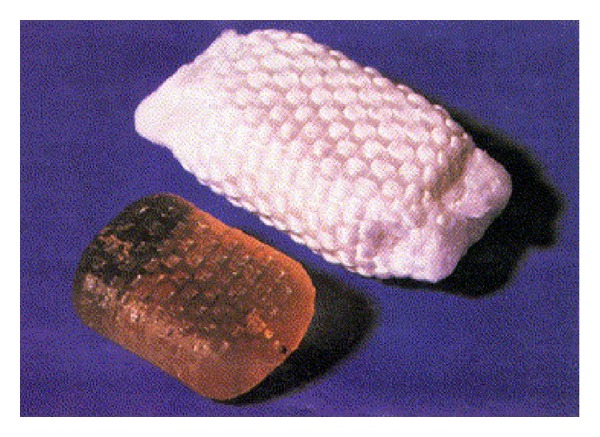
Nucleus replacement, PDN SOLO.

**Figure 9:**
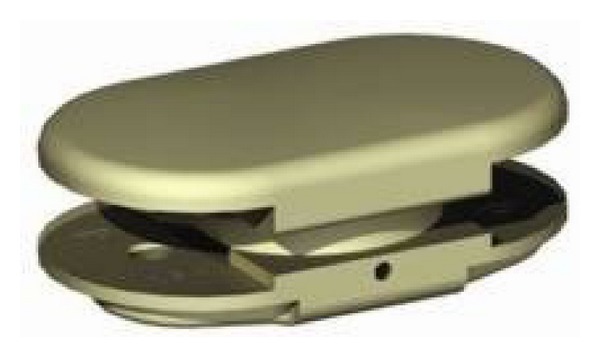
Nucleus replacement, NUBAC.

Subcategories of nucleus replacement was classified by Büttner-Janz, and this classification has been made according to following different criteria [63]. Group A includes injectable, in situ materials, and divided 2 subgroup; (1) *Uncontained* (a) hydrogel adhesive: NuCore and BioDisc (Figures 5 and 6), (b) nonhydrogel nonadhesive: Sinux, (2) *Contained* (a) nonhydrogel: Dascor (Figure 7), PNR, and PDR. Group B includes the preformed implants and divided 2 subgroup; (1) *Nonarticulating* (a) hydrogel: PDN-SOLO (Figure 8), Hydraflex, Neudisc, and Aquarella, (b) nonhydrogel: Newcleus, Neodisc, and Regain, (2) *Articulating* (a) same material of components: NUBAC (Figure 9). Prosthetic disc nucleus (PDN-Raymedica, Inc., Minneapolis, MN) was first implanted in 1996 [64]. This device consists of a polymeric hydrogel pellet surrounded by a high-tenacity polyethylene jacket. The aim was cushioning the intervertebral space for maintaining the function, high and flexibility of the normal disc. It is transformed from the stiff PDN to PDN-SOLO. HydraFlex (Raymedica, Inc., Minneapolis, MN) is the last form of PDN [64]. There are some clinical studies regarding PDN, but the results of studies are not good [65, 66]. It is used limitedly today [4].

NuCore is an injectable nucleus which is adhesive and protein polymer. NuCore is injected percutaneously into the nucleus pulposus as computed tomography guided [63]. NuCore is one of the least stiff materials in this group.

Kaner et al. [4] classified the nucleus replacements in two fashions: (1) nucleus pulposus alternatives that contained the PDN (PDN-Solo, Raymedica, LLC), Nubac (Invibio, Greenville, NC, USA), Daskor (Disc dynamics, Inc., Eden Prairie, Minn), and Neudisc (Replication Medical Inc., New Brunswick, NJ) and (2) Nucleous Pulposus Supports Biodisc (Cryolife, Inc., Kennesaw, Ga), NuCore IDN (Spine Wave Inc., Shelton, Conn), and Gelifex (Gelifex, Inc., Philadelphia, Pa).

## 4. Posterior Dynamic Stabilization 

**Figure 10:**
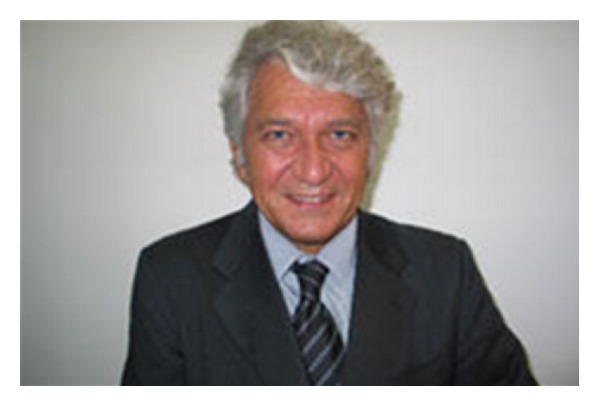
The picture of Henry Graf which used the Graf ligament system.

**Figure 11:**
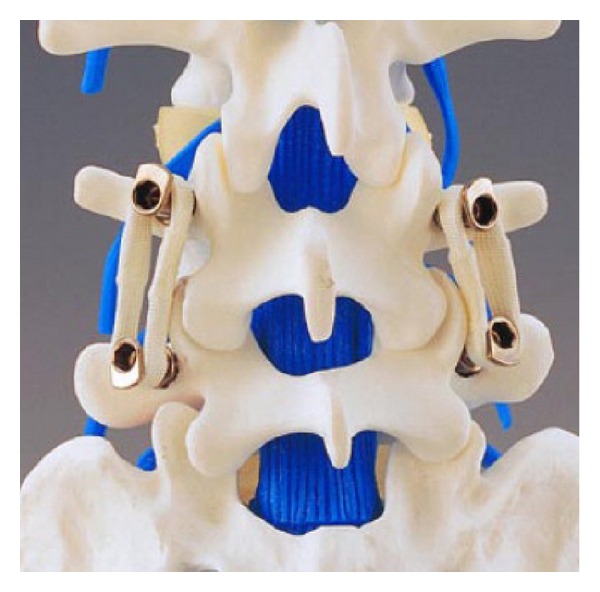
Graf ligament system.

Henry Graf used the Graf ligament system (Sem Co., Montrouce, France) which has been called and designed by him as first posterior dynamic stabilization device (Figures 10 and 11). Graf ligament was developed by Henry Graf opposite to fusion surgeries. This system uses braided polyester bands looped around the screws instead of rods for providing stability while allowing movement. Henry Graf believed that fusion surgeries had some disadvantages and complications when it was used in degenerative diseases of the spine and that Graf ligament would be enough for conditions of the degenerative or chronic instability not overt instability. He suggested that supporting posterior extension band was pretty good in the treatment of degenerative diseases of the lumbar spine. Graf arrived at the achievement with his posterior extention band named Graf ligament as a novel alternative treatment opposite to fusion surgery of the lumbar spine. The concept of Graf's ligament gained popularity primarily in Europe. Graf ligament as posterior extention band was used in condition of chronic instability resulted from degenerative diseases of the lumbar spine. This concept was supported and used [67–71] and found inconvenient [72, 73] by some surgeons in time. Criticism of Graf ligament focused especially on these concerns which are ligament loosening, foramen narrowing, and flat back.

Kanayama et al. reported 10 year follow-up results of posterior dynamic stabilization using Graf artificial ligament [71]. This report was a retrospective long-term study. In this study 56 consecutive patients had artificial Graf ligament, but 43 patients that suffered from degenerative spondylolisthesis (23 patients), disc herniation with flexion instability (13 patients), lumbar spinal stenosis with flexion instability (4 patients), and degenerative scoliosis (3 patients) had sufficient clinical and radiological followup. Patients suffering from degenerative spondylolisthesis and flexion instability improved significantly from symptoms due to low back pain and sciatica, but patients suffering from degenerative scoliosis and/or laterolisthesis had poor clinical improvement. Their long-term results showed that Graf ligamentoplasty was an effective treatment choice for low-grade spondylolisthesis, and flexion instability; however, it has some limitations to correct deformity and is not advocated for the treatment of degenerative scoliosis and/or laterolisthesis.

Choi et al. [74] reviewed retrospectively 43 patients treated with Graf ligamentoplasty for degenerative lumbar stenosis. This study had 8 years follow-up time. They observed angular instability, translational instability, and adjacent segment instability in upper and lower segments, respectively, 28%, 7%, 42%, and 30%. This study shows that Graf ligament can be altered by degeneration of the disc and facet joints at instrumented segments. However, the adjacent segment can be affected because of abnormal load transmission in Graf ligamentoplasty.

**Figure 12:**
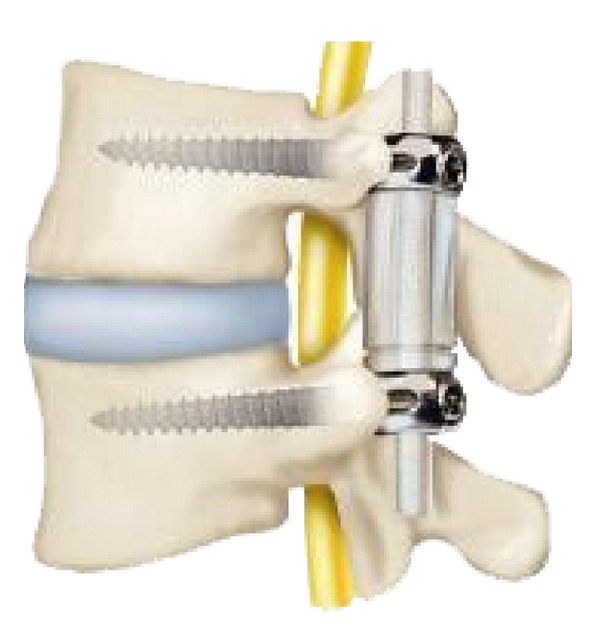
Dynesys posterior dynamic stabilization system.

Dynesys posterior dynamic stabilization system (Zimmer Spine, Inc., Warsaw, IN) is pedicle screw-based system for dynamic stabilization of lumbar spinal segments and was performed first 1994 [64, 75] (Figure 12). Dynesys has cords of polyethylene terephthalate with a tube made from polycarbonate urethane slid over them and fixed to two adjacent pedicle screws [4, 64]. In Dynesys appropriate length spacer is used to control the degree of distraction and compression on the related segment in contrast to Graft soft stabilization system. Therefore Foramen narrowing and flat back syndrome were avoided by using the spacers in Dynesys dynamic system. Dynesys approved by FDA in 2004 for posterior stabilization system as an adjunct to fusion of the lumbar spine [4, 46]. Dynesys was planned to neutralize abnormal forces and restored without pain function to the spinal segments while protecting adjacent segments [46, 76]. Plenty of studies were reported about Dynesys posterior dynamic stabilization system [18, 43, 76–84].

Stoll et al. [78] reported 83 consecutive patients who operated because of lumbar spinal stenosis, degenerative disc disease, disc herniation, and revision surgery. The mean follow-up time was 38.1 months. Their implant-related complications were two screw displacement and screw loosening on radiograph. The one patient of screw displacement was reoperated because of root compression and improved. Just one patient was reoperated because of the loosening of two bilateral screws, and screws were removed and have not been put again. Besides there were 9 complications unrelated to the implant. Seven patients had adjacent segment degeneration and have been reoperated. Mean Oswestry score was 55.4% preoperatively and went down to 22.9% postoperatively. This improvement was found statistically meaningful (*P* < 0.01). Authors suggested in this study that Dynesys was less invasive and theoretically produced less degeneration of adjacent segments.

Putzier et al. [77] reported the compared nucleotomy procedure for the surgical treatment of the lumbar disc prolapse without or with posterior dynamic stabilization with Dynesys. 84 patients underwent nucleotomy procedure and Dynesys was carried out to 35 of them. There were MODIC 1 disc degeneration signs in all patients. The mean follow-up duration was 34 months. This study showed that the patients with additional stabilization with Dynesys revealed meaningful less signs of progressive degeneration.

Schaeren et al. reported 26 consecutive patients suffering from lumbar spinal stenosis and degenerative spondylolisthesis [85]. They performed decompression and posterior dynamic stabilization with Dynesys. Their mean follow-up duration was 52 months. Patients were evaluated clinically and radiographically during followup. Patients satisfaction was obtained high as 95%. Implant failure screw loosening was observed in 3 patients in 2 years after operation; however, nobody was reoperated related for that. 2 of these patients were asymptomatic and other had low back pain. They observed one instability related to a screw breakage in a patient and adjacent segment degeneration in 9 patients (47%) after four years. 

Schnake et al. [18] reported in their prospective clinical study a total of 26 patients with lumbar spinal stenosis with degenerative spondylolisthesis who underwent interlaminar decompression and dynamic stabilization with the Dynesys posterior dynamic system. They concluded that Dynesys maintains enough stability to prevent further progression of instability. Otherwise they mentioned that Dynesys stabilization system does not need using bone Grafting.

Some studies were done regarding the effects of Dynesys on adjacent segments. Schmoelz et al. [86] reported in an in vitro study that Dynesys provided substantial stability while allowing more movement in the stabilized segment in degenerative spinal disorders, and therefore it is considered as an alternative method to fusion surgery. On the other hand, adjacent segments appear to be not influenced by the stiffness of the fixation procedure. Cakir et al. researched adjacent segment mobility after rigid and semirigid instrumentations of the lumbar spine [81]. They study included 26 patients with low back pain and neurogenic claudication due to L4-L5 degenerative instability and spinal stenosis. Patients operated either with decompression and Dynesys posterior stabilization (*n* = 11) or with decompression and fusion (*n* = 15). Range of motion was evaluated at L4-L5 which is index level and adjacent segments which are L3-L4 and L5-S1. They obtained that monosegmental dynamic stabilization along with Dynesys has no beneficial effect on adjacent segment mobility compared with monosegmental fusion and instrumentation. Kumar et al. [80] reported in their prospective case series including 32 patients who underwent just posterior dynamic stabilization with Dynesys (*n* = 20) and additional fusion at one or more levels that disc degeneration at the bridged and adjacent segment seems to continue despite Dynesys dynamic stabilization.

Grob et al. [79] revealed the clinical experience with Dynesys stabilization system. This study was composed of retrospective 50 consecutive case series. All of them instrumented with Dynesys. 31 patients were followed-up with questionnaire at least 2 years. Their result showed that quality of life and improvements in functional capacity were just moderate. Around 50% patients stated that the operation helped or helped a lot. There was no superiority of Dynesys system compared with fusion surgery.

**Figure 13:**
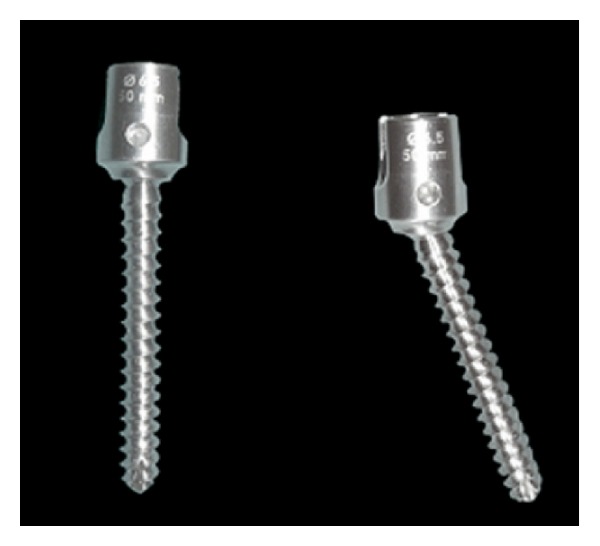
Saphinas dynamic screw.

Cosmic semirigid posterior dynamic system is composed of dynamic screw and rigid rod. Dynamic screw was first used and accepted as a new concept by Strempel in 1999 [47]. Strempel built a dynamic screw with a hinge placed between the head and body of the screw (Cosmic, Ulrich AG, Germany) [47, 50, 87]. Posterior dynamic transpedicular hinged-screw along with rigid rod system enables potential sagittal movement between the screw head and the screw body. This system allows limited motion, which occurs between hinged screw head and the longitudinally placed rod, during flexion-extention behavior of the spine. Cosmic transpedicular dynamic system provides the load sharing on bridged segment, and part of the load placed on the spine is transferred by the system, hereby the effect of the stress shielding on the bones reduced [50, 86, 87]. Bozkuş et al. showed that dynamic stabilization provides a stability that is similar to that provided by rigid systems and that the hinged-dynamic screws allow less stress shielding than standard rigid screws in their in vitro biomechanical study which had used the dynamic/hinged pedicular screw-rod system [88]. Another hinged transpedicular screw-rigid rod system is from Turkey and its name is Safinas Dynamic Screw (Medikon, Turkey) (Figure 13). Safinas hinged screw works similarly to cosmic screw and allows the limited motion in flexion and extention behavior of the spine, but it controls displacement rotation and translation. There are some both clinical [37, 44, 47–50, 52, 66, 89] and biomechanical [25, 39, 86, 88] studies about dynamic screw-rod stabilization system.

Kaner et al. [37] reported the compared study of dynamic stabilization with cosmic dynamic screw-rod and posterior rigid transpedicular stabilization with fusion to treat degenerative spondylolisthesis. This clinical and radiological studies were conducted between 2004 and 2007 and contained totally 46 patients. Twenty-six patients operated via cosmic posterior dynamic stabilization were followed-up at average of 38 months, and fusion group with rigid stabilization that included twenty patients was followed-up at average of 44 months. There were similar results in both groups as a result of VAS, Oswestry, the measurements of lumbar lordosis and segmental lordosis angle after two years of followup. On the other hand, intervertebral space ratios in the cosmic posterior dynamic stabilization group were obtained to be statistically meaningful higher than those in the fusion group. In conclusion of this study it was thought that the disc distance is maintained and disc degeneration is slowed down after using posterior dynamic transpedicular stabilization.

Another compared study of safinas lumbar pedicular dynamic screw-rod and fusion was done by Ozer et al. [48]. Equivalent relief of pain and maintenance of sagittal balance were seen compared with safinas lumbar pedicular dynamic screw-rod and standard rigid screw-rod fixation.

**Figure 14:**
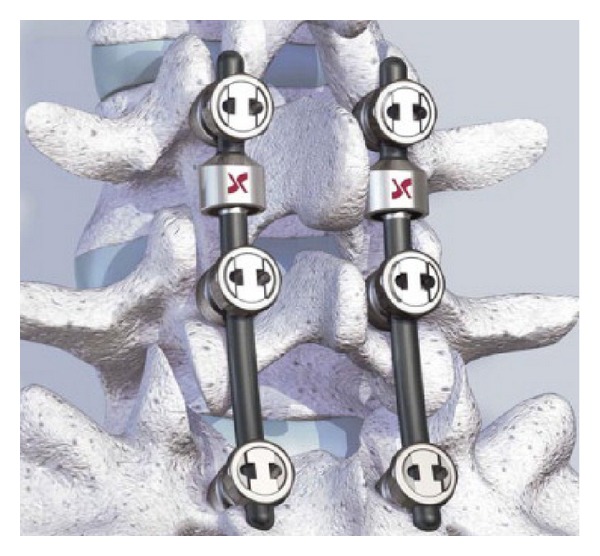
The Isobar TLL dynamic rod.

The Isobar TLL Dynamic Rod (Scient'x, Maitland, FL) is composed of TiAIV alloy and attached between rigid screws (monoaxial or polyaxial) [64] (Figure 14). The Isobar semirigid spinal system (Scient'X's Isobar) received FDA clearance for using as an adjunct to lumbar fusions in November 1999. Isobar TLL semirigid rod allows some limited movement in fusion place. It is aimed by manufacturer that it can promote fusion ratio on the instrumented segment and decrease adjacent segment degeneration via protecting the adjacent disc from excessive stress [90]. Zhang et al. studied the effectiveness of ISOBAR TTL semirigid dynamic stabilization system in treatment of lumbar degenerative disease [91]. This study was done between 2007 and 2011 on 38 patients which are treated because of lumbar degenerative disease. The mean follow-up duration in this study was 27.8 months. The results of this study showed that Isobar TLL had reliable fixation, and no loosening, breakage, and adjacent segment degeneration. Authors suggested that Isobar TLL had good short-term effectiveness in treatments of lumbar degenerative disease. Another biomechanical study using cadaveric human lumbar spine reported that Isobar TLL device may stabilize only the anterior colon [92].

**Figure 15:**
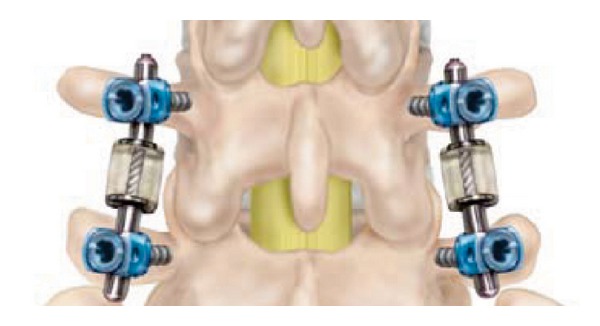
The rod of the CD Horizon Agile.

The rod of the CD Horizon Agile (Medtronic Sofamor Danek, Memphis, TN) is dynamic stabilization device [4, 64] and was used as single level and adjacent to fusion (Hybrid) in two forms (Figure 15). It was developed for using along with rigid rods in 2007 [44]. Dynamic Agile rod was deformed due to overloading and stress in clinical usage in time. As a result it has been removed from the market and terminated of its production [44]. It is the first study that has been done utilizing dynamic rods with dynamic screws in treatment of chronic instability [44]. CD Horizon Agile used Safinas dynamic screws, and good clinical results were observed in this clinical study [44].

**Figure 16:**
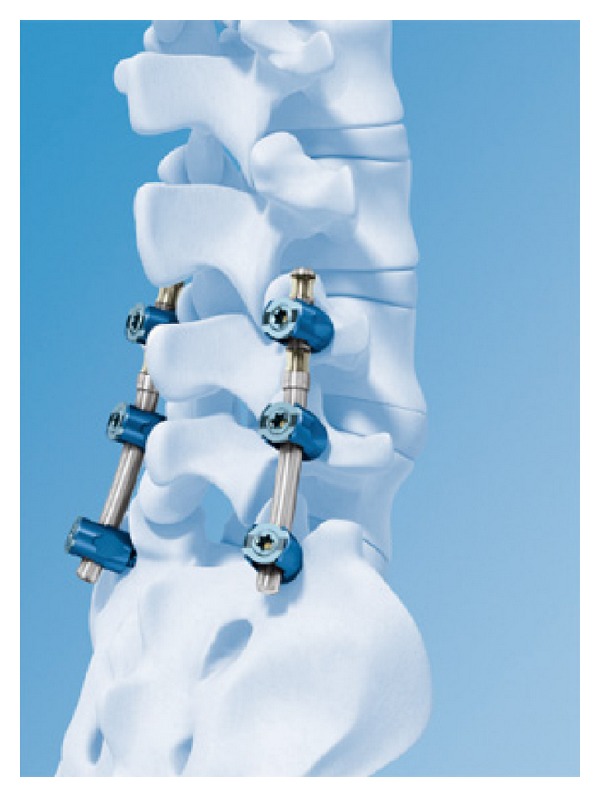
NFlex dynamic stabilization system.

NFlex (N spine, Inc., San Diego, CA) is a dynamic stabilization system [64] (Figure 16). It is composed of two parts: polyaxial rigid screw and titanium and polycarbonate urethane rod. NFlex is first implanted in 2006. A multicenter study was performed related to NFlex dynamic stabilization system [93]. In this retrospective study 72 consecutive patients who have degenerative diseases of the spine underwent surgery with NFlex dynamic system. Mean followup was 25,6 months. VAS and Oswestry disability index of the patients were improved obviously after operations. Just three implant-related complications were observed. This study showed that NFlex dynamic system seems to improve pain and functional scores and may be considered a good alternative to rigid fusion [93].

AXIENT dynamic stabilization system (Innovative Spinal Technologies, Mansfield, MA) is composed of rigid pedicle screws and articulates CoCr sliding rods with a part for depressing during extention which is made of carbonate urethane [64]. This system permits segmental motion provided to avoid excessive motion in flexion, extention, and axial rotation.

Accuflex rod system (Globus Medical Inc.) is a semirigid rod which has been situated between rigid rods. Accuflex system obtained FDA clearance in 2005 as a single-level tool to stabilize lumbar interbody fusion [46]. A clinical study was done related to Accuflex rod system. This study reported that Accuflex semirigid system showed clinical benefits and ceased the degenerative process in 83% of the patients although high incidence of implant failure (22.22%) was observed [94].

**Figure 17:**
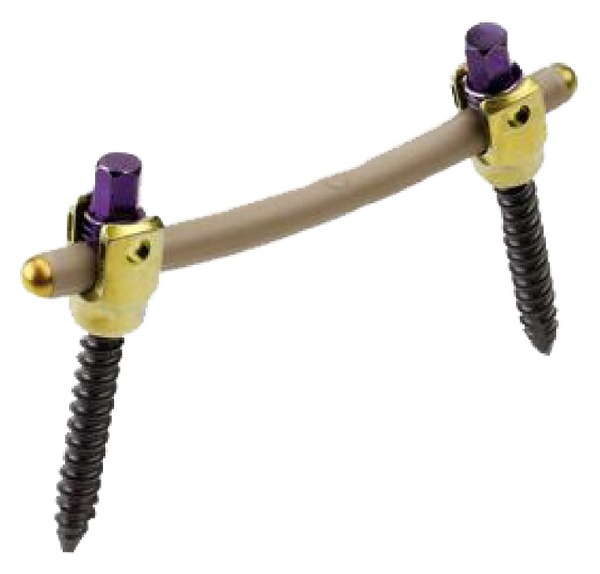
CD horizon legacy peek rod.

CD Horizon legacy peek rod (Medtronic, Safamor Danek, Memphis, TN) has been introduced to the market as a semirigid alternative to titanium rods (Figure 17). FDA clearance has been got in June 2005. In a biomechanical study Gornet et al. reported that peek rod system provided intervertebral stability comparable to currently marketed titanium lumbar fusion constructs [95]. Ormond et al. studied retrospective 42 case series from 2007 to 2009 for degenerative lumbar disease and performed them posterior lumbar fusion using PEEK rods [96]. They observed that 8 of 42 patients with PEEK rods underwent reoperation. Reoperations included adjacent segment degeneration (5/8) and nonunion with cage migration (3/5). In conclusion authors reported that PEEK rods demonstrated similar fusion and reoperation rate in comparison with other instrumentation modalities.

**Figure 18:**
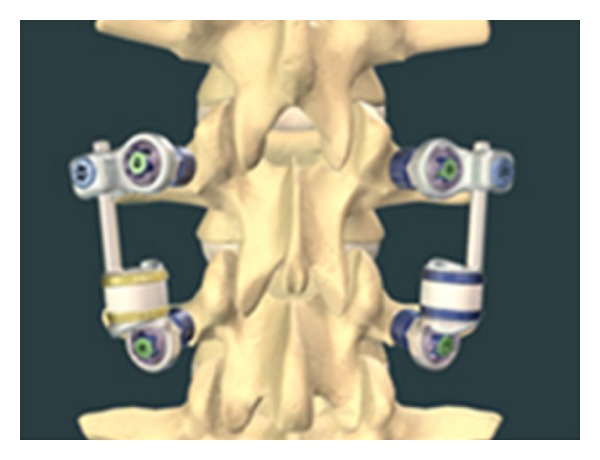
The Stabilimax NZ which is a pedicle-based posterior dynamic system.

The Stabilimax NZ (Applied Spine Technologies, New Haven, CT) is a pedicle-based posterior dynamic system which was developed and designed to specifically address pathological alterations in the neutral zone by Panjabi [97, 98] (Figure 18). Its indications include moderate to severe degenerative lumbar spinal stenosis and discogenic low back pain. The Stabilimax NZ is composed of a system of ball and socket joints to decrease the load on the pedicle screw and double connecting springs [46, 97, 98].

## 5. Total Facet Replacement Systems

**Figure 19:**
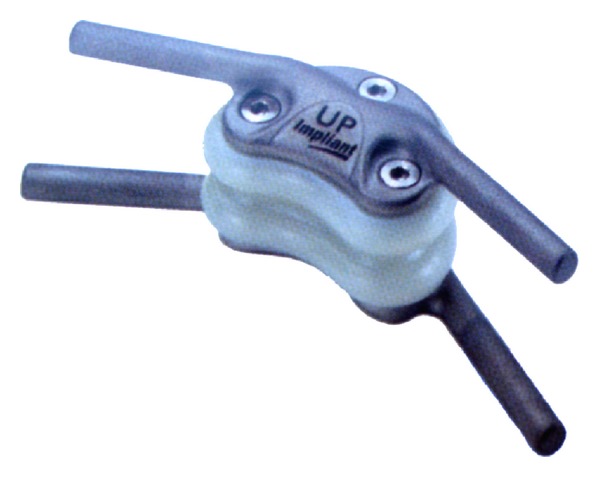
The total posterior arthroplasty system (TOPS).

**Figure 20:**
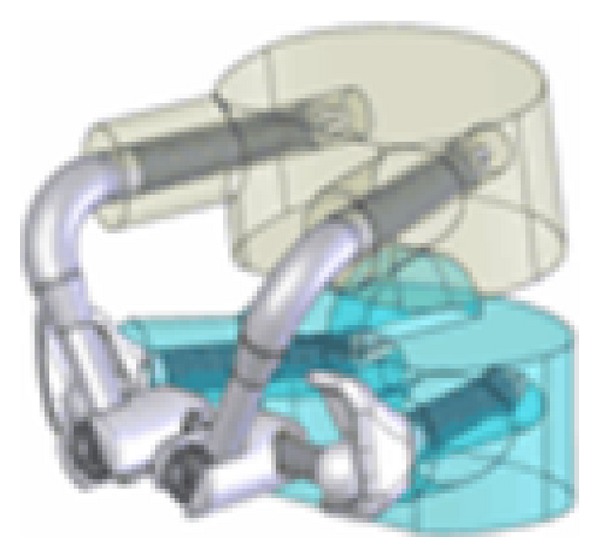
The total facet arthroplasty system (TFAS).

Total facet replacement systems are designed to totally restore facet joints functionally. The degeneration of facet joints generally results from intervertebral disc degeneration, because of this reason facetogenic pain can occur along with severe disc degeneration, and it may be used in significant facet and intervertebral disc degeneration either alone or with total disc arthroplasty [45]. Total facet replacement systems can be used at the situation of reconstruction of the spine due to iatrogenic facetectomy [45, 64]. There are some total facet replacement systems such as the total facet arthroplasty system (TFAS), the total posterior arthroplasty system (TOPS) (Figure 19) and anatomic facet replacement system (AFRS) [4, 45, 46, 64]. TFAS (Archus Orthopedics, Inc., Redmond, WA) completely restores the total joints and is totally made of metal [64] (Figure 20). It is nonfusion spinal implant and is developed for severe facet degeneration with lumbar spinal stenosis. It needs total laminectomy and facet resection for the implantation of TFAS. TOPS (Impliant Spine, Princeton, NJ) is composed of metal and plastic material and a pedicle screw-based systems [45, 46, 64]. Posterior facets and lamina are resected totally, and nonfusion TOPS device, which enables physiologic range of motion, is implanted. It is thought that TOPS preserves motion, prevents abnormal load sharing at both adjacent and treated segments, and restores the neutral zone [99]. Nowadays there are several clinical and biomechanical studies on total facet replacement systems, and similar facet replacement systems having the same properties have been developed for clinical usage [45].

## 6. Posterior Interspinous Spacer Devices

**Figure 21:**
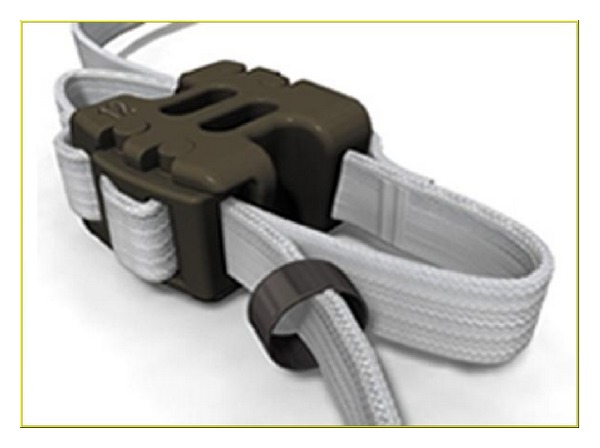
Wallis posterior interspinous spacer device.

**Figure 22:**
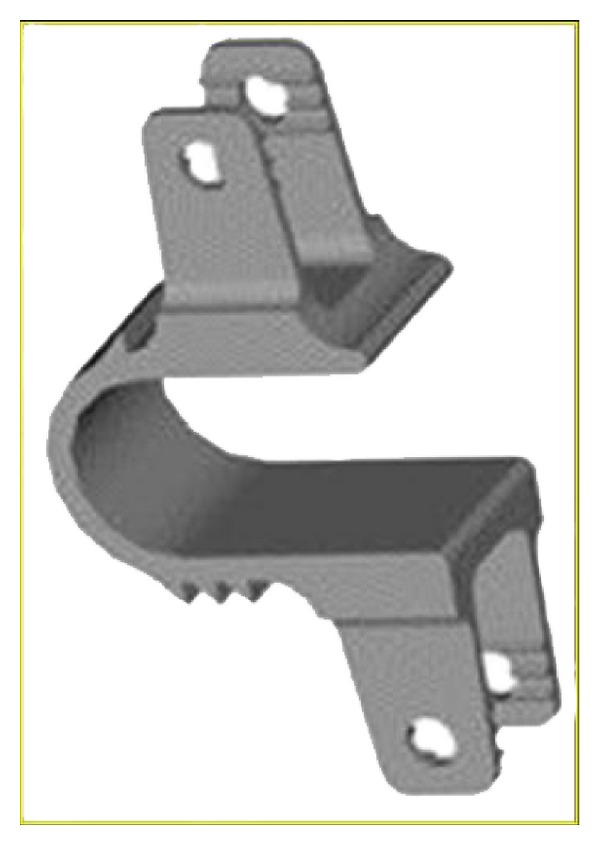
Coflex posterior interspinous spacer device.

**Figure 23:**
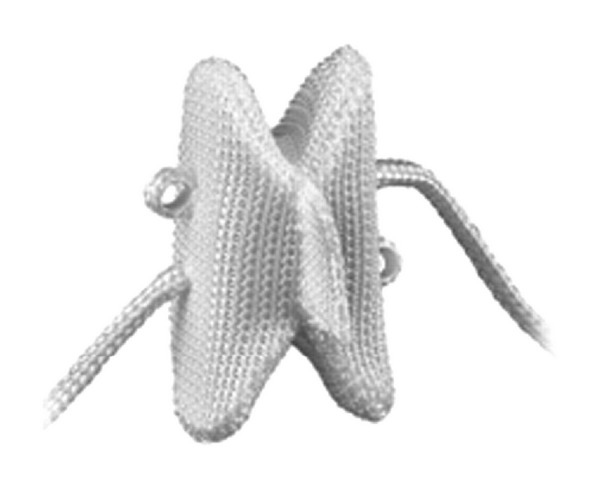
The device for intervertebral assisted motion (DIAM).

**Figure 24:**
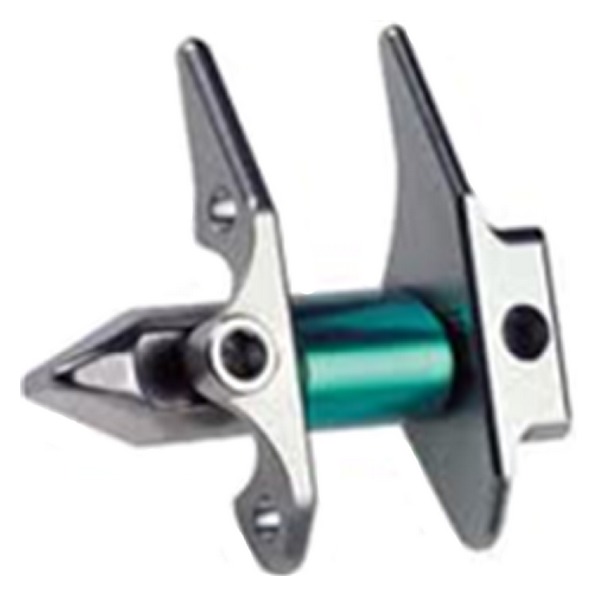
X-STOP posterior interspinous spacer device.

Lumbar interspinous spacer devices have recently been popular for alternative treatment of lumbar degenerative diseases. Interspinous spacer devices are used in lumbar spine from L1 to L5 for treatment of central spinal canal stenosis with neurogenic claudication, foraminal stenosis, facet joint disease, and the dorsal disc anloading in extention [4, 64]. One of the first interspinous spacer devices has been developed for lumbar stabilization in 1986 and was called as wallis system [100]. Wallis system (Abbott Spine, Inc., Austin, TX) consists of two parts: these are PEEK and two woven polyester bands (Figure 21). The first implant was developed, and in 2002 a second generation of the wallis implant has been produced [64, 101]. Senegas et al. who developed the wallis device reported the clinical evaluation of the wallis interspinous spacer device with a 13-year mean followup. They reported 107 patients who filled out the health questionnaires. All patients had schedule for fusion surgery because of lumbar canal stenosis and lumbar disc herniation, or both. While 87 patients have still interspinous spacer today, other 20 patients experienced implant removing and had reoperation as fusion. They concluded that wallis provided good clinical results at last 13 years and 80% of patients were protected from fusion surgery and living now with posterior dynamic stabilization. Some similar systems have been produced and offered to the market. Some of them are Coflex (Paradigm Spine, LLC, New York, NY) (Figure 22), the device for intervertebral assisted motion (DIAM) (Medtronic Sofamor Danec, Memphis, TN) (Figure 23), the Fulcrum-assisted soft stabilization (FASS), The superion spacer (VertiFlex Inc., San Clemente, CA), and X-STOP (Kyphon, Inc., Sunnyvale, CA) (Figure 24) [4, 64, 101]. Kabir et al. [102] studied on a systematic review of clinical and biomechanical evidence about lumbar interspinous spacers. They reported that the biomechanical studies with all the devices showed that interspinous spacer devices have a beneficial effect on the kinematics of the degenerative spine. They also mentioned that Lumbar interspinous spacer devices may have a potential beneficial effect in selected group of patients with degenerative disease of the lumbar spine. A new biomechanical study about X-STOP showed that implantation of the X-Stop devices can effectively distract the interspinous process space at the diseased level without causing apparent kinematic changes at the adjacent segments during the studied postures [103]. In other study being randomized, controlled, prospective multicenter trial Zucherman et al. suggested that the X-STOP provides a conservative yet effective treatment for patients suffering from lumbar spinal stenosis and the X-STOP may be alternative treatment to both decompressive spine surgery and conservative treatment [104].

## 7. Conclusion

Nowadays in market different both anterior and posterior dynamic stabilization devices of the lumbar spine are found. Various both biomechanical and clinical studies have been made about dynamic stabilization systems of the lumbar spine. Recently finite element studies also have been begun more and more on these systems. Dynamic stabilization systems of lumbar spine on rigid stabilization have some advantages such as increased load sharing and controlled motion without cutting off the stability which could be an important factor in decreasing adjacent segment degeneration, but this matter has not been yet proved clearly. In the future it needs prospective compared clinical studies for providing the benefit of dynamic stabilization systems.
